# Fidelity to motivational interviewing and subsequent cannabis cessation among adolescents three months after brief intervention

**DOI:** 10.1186/1940-0640-7-S1-A8

**Published:** 2012-10-09

**Authors:** Jim McCambridge, Maria Day, Bonnita Thomas, John Strang

**Affiliations:** 1 Center for Research on Drugs and Health Behavior, Department of Public Health and Policy, London School of Hygiene and Tropical Medicine, London, UK; 2 National Addiction Center, Institute of Psychiatry, King's College London, UK

## 

This study tested whether differences in cannabis cessation three months after a single session of motivational interviewing (MI) may be attributable to fidelity to MI. All audiorecordings with necessary three-month follow-up data (N = 75) delivered by four individual practitioners within a randomized controlled trial (RCT) were used. Participants were weekly or more frequent cannabis users aged 16–19 years old in further-education colleges. All tapes were coded with the Motivational Interviewing Treatment Integrity (MITI) scale, Version 2, by two coders. Satisfactory inter-rater reliability was achieved. Differences between and within practitioners in fidelity to MI were consistently detected. After controlling for practitioner effects, MI spirit and the proportion of complex reflections were independently predictive of cessation outcome. No other aspects of fidelity were associated with outcome. These two particular aspects of enhanced fidelity to MI were predictive of subsequent cannabis cessation three months after a brief intervention among young cannabis users.

